# Management and outcomes of ocular surface squamous neoplasia at a tertiary hospital, South Africa

**DOI:** 10.1038/s41433-025-03926-8

**Published:** 2025-07-25

**Authors:** Roland Höllhumer, Pamela Michelow, Elena Libhaber, Susan Williams

**Affiliations:** 1 Department of Neurosciences, Division of Ophthalmology, University of the Witwatersrand, Johannesburg, South Africa; 2 Cytology Unit, National Health Laboratory Service and Department of Anatomical Pathology, Faculty of Health Sciences, University of the Witwatersrand, Johannesburg, South Africa; 3 Health Sciences Research Office, Faculty of Health Sciences, University of the Witwatersrand, Johannesburg, South Africa

**Keywords:** Conjunctival diseases, Eye cancer

## Abstract

**Aims:**

The aim of this study is to describe the outcomes of OSSN management at a tertiary eye hospital in Johannesburg, South Africa.

**Methods:**

This was a prospective interventional study, which included patients presenting with conjunctival masses at a tertiary eye hospital from December 2019 to February 2022. An electronic questionnaire was completed to document demographic data, presenting history, and associated risk factors. All patients had a biopsy to confirm the diagnosis. Tumours that occupied less than or equal to 4 limbal clock hours had an excision biopsy. Larger tumours had an incision biopsy followed by topical 5-fluorouracil (5FU) as primary management. Recurrences and positive margins were managed with 5FU. Patients were followed up for 2 years to monitor for recurrence.

**Results:**

One hundred and nine patients with 114 conjunctival masses were included in this study. Ninety-four percent (107/114) of patients had an excision biopsy as their primary management, with a recurrence rate of 0.9% (1/107). Seven patients had 5FU as primary therapy with a complete resolution rate of 71% (5/7) and a recurrence rate of 14% (1/7).

**Conclusion:**

A standardised management approach for OSSN based on the size of the tumour resulted in high resolution rates and a low rate of recurrence.

## Introduction

Ocular surface squamous neoplasia (OSSN) is the most common ocular surface tumour with incidence rates of 0.03–1.9 per 100,000 persons/year in high income countries and 1.6–3.4 per 100,000 persons/year in low-income countries [1]. The main associated risk factors include ultraviolet-B radiation, HIV infection and human papillomavirus (HPV) infection [1].

**Fig. 1:**
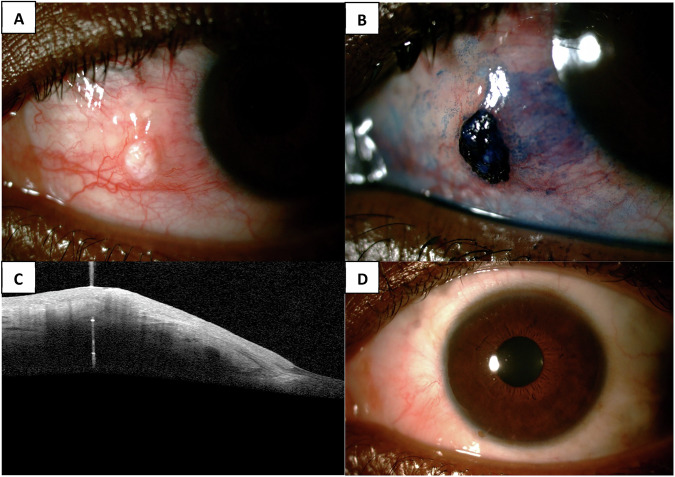
Conjunctival intraepithelial neoplasia 3. **A** Anterior segment photo, **B** Methylene blue stain, **C** anterior segment optical coherence tomography, **D** anterior segment photo after surgical excision with 4 mm margins and double freeze thaw cryotherapy.

The diagnosis is made clinically, with further testing done to confirm the diagnosis (Fig. 1). These include biopsy with histology, cytology, anterior segment optical coherence tomography (AS-OCT), confocal microscopy and methylene blue stain [2, 3]. On histology OSSN can be divided into conjunctival intra-epithelial neoplasia (CIN), squamous cell carcinoma in-situ (CiS) and invasive squamous cell carcinoma (SCC). CIN can be further stratified into three grades according to the degree that dysplastic cells occupy the epithelium [2].

Management of OSSN can be divided into two main groups, surgical and medical [2]. Surgical management has been the primary management approach traditionally. It is indicated when four or less limbal clock hours are involved and when the basal tumour diameter is less than 15 mm [4]. This ensures that the main complication from surgery, limbal stem cell failure, is avoided. Other complications after surgery include infection, scarring, persistent epithelial defect and pyogenic granuloma [4, 5]. The no-touch surgical technique popularised by Shields et al. [4] is the preferred surgical approach, together with double freeze-thaw cryotherapy of the conjunctival margins and limbus. Recurrence rates after surgical excision vary according to the surgical approach and adjuvant methods employed. In the absence of cryotherapy, recurrence rates can be as high as 28.5%, but are reduced to 7.7% with the use of cryotherapy [6]. Other adjuvant therapies that are used to manage the surgical margins include medical therapy and brachytherapy [7].

With the advent of less invasive diagnostic modalities, there has been a progressive move towards medical therapy as primary, neoadjuvant or adjuvant therapy [8]. These include interferon-α2b (IFN), 5-fluorouracil (5FU) and mitomycin-C (MMC) [7]. IFN is an immunotherapy that is considered the best of these three options due to a low side effect profile, high resolution rates and low recurrence. It is unfortunately no longer available in most countries. 5FU, a pyrimidine analogue, has an intermediate surface toxicity profile with similar efficacy to IFN and MMC. MMC, an alkylating agent, has been reported to have the highest surface toxicity with risks of limbal stem cell deficiency [7]. Medical therapy has resolution rates ranging from 76 to 100%, with no significant difference between the three options [7]. Recurrence rates range from 0 to 35%, which is in a similar range to surgical excision [7].

Advanced disease not amenable or not responding to the above treatment options may need more extensive surgery in the form of an enucleation or exenteration [7].

There is a high incidence of OSSN in South Africa and well-established management guidelines reported in the literature. There is however limited data on the efficacy of OSSN management in the South African setting. The aim of this study is to describe the outcomes of OSSN management at a tertiary eye hospital in Johannesburg, South Africa.

## Materials and methods

This prospective interventional study was conducted at a tertiary eye hospital in Johannesburg, South Africa. Ethics approval was granted by the Human Research Ethics committee of the University of the Witwatersrand (M190729) and adhered to the tenets of the declaration of Helsinki. The clinical study is registered with the Pan African Clinical Trials Registry (PACTR201912900667480).

The study included patients that presented with conjunctival masses between December 2019 and February 2022 (Supplement 1). Recruitment took place during the COVID19 pandemic. Patients were recruited consecutively in between COVID19 waves if they had conjunctival masses that were either considered to be suspicious for OSSN or were considered benign but remained symptomatic despite medical therapy (topical lubricants and/or corticosteroids). Features of OSSN included a raised lesion with feeder vessels, a mass with leukoplakia, pigmented mass, and a diffuse pearly lesion extending from the conjunctiva onto the cornea. Exclusion criteria for enrolment included age less than 18 years; females that were pregnant or breast feeding; a history of previous topical chemotherapy or surgery in the involved eye; lesions with a basal diameter of greater than 15 mm or where adjacent structures other than the cornea or sclera were involved; conditions that prevented performing study investigations; the presence of conditions that are known to predispose to OSSN (xeroderma pigmentosum); or a diagnosis of primary acquired melanosis.

Patients that met the inclusion criteria underwent informed consent and completed an interview to document demographic data, history of presenting complaint and the presence of associated risk factors (HIV status, UV exposure history, smoking, immunosuppressive conditions or medication, ocular surface inflammation, ocular injury, regular exposure to petroleum products). A clinical examination and anterior segment photography were performed with slit lamp to document clinical features. OCT and methylene blue stain were performed at the initial visit, with impression cytology and biopsy for histology at the time of surgery.

All patients had a biopsy to confirm the diagnosis by histology (Supplement 2). Masses suspicious of OSSN that occupied less than or equal to four clock hours of the limbus had an excision biopsy with 4 mm margins using the Shields no touch technique [4] and double freeze-thaw cryotherapy to the limbus and free conjunctival edge. Alcohol assisted keratoepitheliectomy with at least 2 mm margins was performed if there was extension onto the corneal surface and a lamellar sclerectomy with 2 mm margins was performed if there was scleral invasion. Excision biopsies were mounted on a sponge with orientation sutures. Margins other than the corneal margin were assessed for radial and deep involvement. The corneal margin was not included as it was routinely managed with alcohol assisted keratoepitheliectomy and cryotherapy at the time of surgery. If margins (radial and/or deep) were positive on histology, one cycle of 5FU was given once the epithelium had healed. Brachytherapy was not routinely available for the management of deep margins.

Larger lesions had an incision biopsy performed to confirm diagnosis and received topical chemotherapy in the form of 5FU. One cycle of 5FU included one month of 5FU 1% dosed four times a day, followed by a rest month. Patients received treatment until resolution and then for one cycle after resolution. All patients also used prednisolone acetate 1% twice a day and a lubricating ointment four times a day to minimise the side effects of topical 5FU.

Lesions were considered resolved when both the clinical and OCT examinations were normal. Patients were followed up for 24 months after resolution (3, 6, 9, 12, 18 and 24 months) to monitor for recurrence, complications of surgery and side effects of 5FU treatment. A standardised data collection sheet designed for this study was used at all follow-up visits. If a participant was initially lost to follow-up and then returned for review while the study was still ongoing, the visit and follow up time was captured. If recurrence was noted, topical 5FU was used as first line therapy and dosed as above. Patients who did not have resolution on 5FU either had further surgery or brachytherapy with ruthenium-106. Surgery included either excision as described above, or enucleation/exenteration if too advanced. In bilateral cases, the patients were followed up for 24 months after resolution of the second eye.

Conjunctival lesions that were clinically considered benign had a simple excision and conjunctival autograft. If these lesions showed OSSN on histology, margins were reviewed to assess the need for an adjuvant cycle of 5FU and patients were followed for recurrence as above.

Data analysis was performed using STATA (StataCorp LLC, Texas, USA), version 17.0. Descriptive statistics were used for patient characteristics, clinical features, associated risk factors, management, outcomes, complication of surgery and side effects of 5FU therapy. Each tumour was assessed independently. Shapiro-Wilk test was used to test for normality on continuous variables. Categorical data are presented as numbers and percentages. Continuous data that do not show a normal distribution are summarised with medians and interquartile range. Fisher exact was used to compare recurrence rates between medical and surgical therapy and rank sum test was used to compare follow-up times between these two groups. A significance level of <0.05 was used.

## Results

One hundred and nine patients with 114 conjunctival masses were included in this study (Table 1). The median age was 45 years (IQR: [36–53]), with 54% female. The histology profile included 84% CIN (96/114), 5% CiS (5/114) and 11% SCC (13/114). The main risk factor was HIV infection with 70% (80/109) of the participants infected. There were five patients with bilateral disease. Four were female and they had a mean age of 42 years (SD: 9.7) (Table 2, Supplement 3).

**Table 1:** Demographics, signs, histology and risk factors for participants and tumours.

Demographics (*n* = 109)	
	*n*
Median age in years [IQR]	45 [36–53]
Sex (%)	
Male	50 (46)
Female	59 (54)
Race (%) (*n* = 109)	
Black African	111 (97)
Mixed Race	3 (3)
Associated Risks (*n* = 109)	
Declined HIV testing	3 (3)
HIV positive (%)	80 (70)
Tobacco use (%)	21 (18)
Median hours of sun exposure [IQR]	3 (2-8)
Ocular trauma (%)	3 (3)
Chronic ocular surface inflammatory disease (%)	2 (2)
Petroleum exposure (%)	1 (1)
Median vitamin A levels^a^ [IQR]	1.64 [1.43 – 2.11]
Hepatitis B (%)	7 (6)
Hepatitis C (%)	0
Signs (*n* = 114)	
Mass location (%)	
Epicentre	
Conjunctiva	56 (49)
Limbus	58 (51)
Cornea	
Quadrants (%)	
Nasal	95 (83)
Temporal	22 (19)
Superior	3 (3)
Inferior	8 (7)
Clinical features (%)	
Vascularised	100 (88)
Elevated	101 (89)
Leukoplakia	61 (54)
Pigmented	58 (51)
Mild (0–33%)	24 (41)
Moderate (34–66%)	16 (28)
Severe (67–100%)	18 (31)
Morphology (%)	
Fibrovascular	14 (12)
Nodular	4 (4)
Diffuse	2 (2)
Placoid	95 (83)
Leukoplakic	60 (63)
Gelatinous	35 (37)
Papilliform	10 (11)
Median surface area, mm^2^ [IQR]	20 [9–29]
Median limbal clock hours involved [IQR]	2 [1–3]
Tumour thickness (%)	
<1.5 mm	14 (12)
=1.5 mm	82 (72)
>1.5 mm	18 (16)
Scleral fixation on examination (%)	13 (11)
Histology (*n* = 114)	
CIN 1	35 (31)
CIN2	28 (25)
CIN 3	33 (29)
CiS	5 (4)
SCC	13 (11)

Vitamin A, hepatitis B and hepatitis C were done for 66 cases.

*IQR* interquartile range, *CIN* conjunctival intra-epithelial neoplasia, *CiS* squamous cell carcinoma in-situ, *SCC* squamous cell carcinoma, *HIV* human immune-deficiency virus.

^a^All vitamin A levels below normal had CRP reviewed to exclude a false low level due to the acute phase of an infection (none had this). Normal range for vitamin A is 1.05–2.8 umol/L.

**Table 2:** Summary of demographics, tumour characteristics, risk factors, histology, management and outcomes of bilateral cases.

	Eye	Delay in presentation between eyes	Demographics	Tumour characteristics	Risk factors	Histology	Initial management	Outcome
1	L		33 year old black female	Gelatinous <1.5 mm thick 2 limbal clock hours 42.5 mm^2^ surface area	HIV+, on ARVs VL: 148 000 CD4: 179	CIN 3	Surgical excision with 4 mm margins, alcohol epitheliectomy, double freeze-thaw cryotherapy	35 months of follow-up with no recurrence
	R	No delay		Gelatinous <1.5 mm thick 1 limbal clock hour 23.5 mm^2^ surface area		CIN 3	Surgical excision with 4 mm margins, double freeze-thaw cryotherapy	24 months of follow-up with no recurrence
2	R		34 year old black male	Leukoplakic, gelatinous 1.5 mm thick 1 limbal clock hour 38 mm^2^ surface area	HIV+, ARV naïve CD4: 3 Sun exposure	CiS	Surgical excision with 4 mm margins, double freeze-thaw cryotherapy	24 months of follow-up with no recurrence
	L	22 months		Papilliform <1.5 mm thick 4 limbal clock hour 77mm^2^ surface area		CIN 2	Surgical excision with 4 mm margins, alcohol epitheliectomy, double freeze-thaw cryotherapy, 1 cycle of 5FU of positive radial margins	12 months of follow-up with no recurrence
3	R		51 year old black female	Gelatinous <1.5 mm thick 1 limbal clock hour 3.2 mm^2^ surface area	HIV+, on ARVs Virally supressed CD4: 934	CIN 1	Surgical excision with 4 mm margins, alcohol epitheliectomy, double freeze-thaw cryotherapy	26 months of follow-up with no recurrence
	L	No delay		Leukoplakic <1.5 mm thick 1 limbal clock hour 2 mm^2^ surface area		CIN 1	Surgical excision with 4 mm margins, double freeze-thaw cryotherapy	24 months of follow-up with no recurrence
4	R		33 year old black female	Gelatinous <1.5 mm thick 3 limbal clock hours 40.5 mm^2^ surface area	HIV+, on ARVs Virally supressed CD4: 260	CIN 3	Surgical excision with 4 mm margins, alcohol epitheliectomy, double freeze-thaw cryotherapy	41 months of follow-up with no recurrence
	L	12 months		Leukoplakic <1.5 mm thick 1 limbal clock hour 4 mm^2^ surface area		CIN 1	Surgical excision with 4mm margins, alcohol epitheliectomy, double freeze-thaw cryotherapy, 1 cycle of 5FU of positive radial margins	25 months of follow-up with no recurrence
5	R		54 year old black female	Gelatinous >1.5 mm thick 3 limbal clock hour 80.8 mm^2^ surface area	HIV+, on ARVs Virally supressed CD4: 102	CIN 3	Surgical excision with 4 mm margins, alcohol epitheliectomy, double freeze-thaw cryotherapy	14 months of follow-up with no recurrence
	L	No delay		Leukoplakic <1.5 mm thick 1 limbal clock hour 38.5 mm^2^ surface area		CIN 2		22 months of follow-up with no recurrence

*CIN* conjunctival intra-epithelial neoplasia, *CiS* squamous cell carcinoma in-situ, *HIV* human immune-deficiency virus, *ARVs* anti-retroviral therapy.

Ninety-four percent (107/114) of tumours had an excision biopsy as their primary management modality (Table 3). This was combined with adjuvant cryotherapy in 88% (94/107), alcohol epitheliectomy in 46% (49/107) and lamellar sclerectomy in 10% (11/107). The patients that did not have cryotherapy had surgery performed for pterygium, with OSSN as an incidental finding on histology. In these cases, the margins were assessed for the need for adjuvant therapy (5FU). OSSN was found as an incidental finding in 13% (15/114) of lesions removed. Conversely, twelve percent (14/114) of lesions suspicious of OSSN were benign on histology. For all excision biopsies the margins were assessed, with positive margins in 36% (38/107). The radial margins were involved in 84% (32/38) of these and the deep margins in 37% (14/38). Two patients had complications from surgery, one with cicatrisation and one with a persistent epithelial defect (Supplement 4).

**Table 3:** OSSN management and outcomes for surgery and 5-fluorouracil.

Surgical		5-Fluorouracil	
	*n* (%)		*n* (%)
Excision biopsy	107	Primary management	7
Positive margins	38 (36)	Mean cycles^a^ (SD)	2.7 (1.38)
Radial	32^a^ (84)	Partial resolution	2 (29)
Deep	14 (37)	Complete resolution	5 (71)
Alcohol epitheliectomy	49 (46)	Mean time to resolution in months (SD)	6 (2.3)
Lamellar scleral excision	11 (10)	Management of recurrence	2 (2)
Adjuvant cryotherapy	94 (88)	Mean cycles^a^ (SD)	2 (1.4)
Adjuvant 5FU for positive margins	38 (36)	Complete resolution	1 (50)
		Failure	1 (50)
Outcomes			
Median duration of follow-up in months [IQR]	24 [20–24]	Median duration of follow-up in months [IQR]	12 [8–24]
Defaulted before 24 months	28 (26)	Defaulted before 24 months	4 (57)
Recurrence rate	1 (0.9)	Recurrence rate	1 (14)
Time to recurrence months	8	Time to recurrence months	6

*IQR* inter-quartile range, *SD* standard deviation.

^a^positive radial margins in patients with suspected OSSN (*n* = 28) and in patients with pterygia clinically that had OSSN on histology with positive margins (*n* = 4).

Seven patients (6%, 7/114) had medical therapy with 5FU as a primary modality with complete resolution in 71% (5/7)(Table 4). 5FU was used as the first management option in patients with recurrence (2/2), with a mean of 2 cycles used (SD: 1.4)(Table 4). Side effects from topical 5FU were mild and did not result in the cessation of therapy.

**Table 4:** Characteristics, management and outcomes of patients with incomplete tumour resolution on topical 5FU and patients with recurrence of OSSN.

**Incomplete resolution with topical 5FU**						
**Demographics**	**Tumour characteristics**	**Risk factors**	**Histology**	**Initial management**	**Salvage management**	**Outcome**
46 year old black male	Leukoplakic 1.5 mm thick 5 limbal clock hours 105 mm^2^ surface area	HIV+ on ARVs Virally suppressed CD4: 179	SCC	5FU, 4 cycles	Surgical excision of residual tumour, with 2 more cycles of 5FU	26 months of follow-up without recurrence
31 year old black male	Gelatinous, papilliform, pigmented <1.5 mm thick Circumferential, 5 limbal clock hours	HIV+, ARV naïve CD4: 97 AKC	CIN3	5FU, 5 cycles	Surgical excision of residual tumour with alcohol assisted epitheliectomy and 1 further cycle of 5FU	24 months of follow-up without recurrence
**Recurrence after surgical or medical therapy**						
43 year old black female	Diffuse <1.5mm thick 8 limbal clock hours	HIV+, ARV naïve CD4: 145	CIN3	5FU, 2 cycles with resolution	Recurrence at 6 months 5FU, 3 cycles, with resolution	Died 1 month after last review of unknown cause
51 year old black female	Gelatinous >1.5 mm thick 4 limbal clock hours 25 mm^2^ surface area	HIV+, ARV naïve CD4: 176	SCC with positive radial and deep margins	Surgical excision with 4 mm margins, alcohol epitheliectomy, lamellar sclerectomy, double freeze-thaw cryotherapy, 5FU for 1 cycle	Recurrence at 8 months 5FU, 1 cycle, without resolution	Developed intra-ocular extension and had enucleation

*5FU* 5-fluorouracil, *OSSN* ocular surface squamous neoplasia, *HIV* human immunodeficiency virus, *ARVs* anti-retroviral therapy, *SCC* invasive squamous cell carcinoma, *AKC* atopic keratoconjunctivitis, *CIN* conjunctival intraepithelial neoplasia.

The overall median follow-up duration for the study was 24 months (IQR: [18–24]). Twenty-nine percent of patients (32/114) did not complete follow-up to 24 months. The median follow-up for the surgery group was 24 months (IQR: [20–24]) and for the medical group 12 months (IQR: [8–24]) with not enough evidence to show a significant difference between the two (*p* = 0.09). In the surgery group nine patients (9%, 9/107) defaulted in the first year and a further 19 (19%, 19/107) during the second year of follow-up. Two of the patients (29%, 2/7) using 5FU as primary therapy defaulted in the first year of follow-up and another two during the second year of review. There was recurrence in two patients (1.8%, 2/114) during this time, one each from the medical and surgical treatment groups (Tables 3 and 4). One of these resolved with further 5FU treatment and the second developed intra-ocular extension that required enucleation.

## Discussion

OSSN is the most common ocular surface tumour with a high burden of disease in Africa [9]. There is a paucity of data on the management and outcomes of OSSN in the South African context. Our study reports high resolution and low recurrence rates using a standardised management approach determined by the size of the tumour.

The traditional gold standard of management for OSSN is excision with adjuvant cryotherapy[4]. The greatest determinant for recurrence is the tumour margin status and the management thereof. The tumour margin is frequently involved on histology despite employing 2–4 mm macroscopic margins during surgery [10–13]. Recurrence rates have been found to be as high as 56% when margins are involved and no cryotherapy is used, dropping to 33% when margins are clear [14]. Cryotherapy as an adjuvant has decreased recurrence rates from 28.5% to 7.7% in a study that had 66% positive margins after excision [6]. When adding medical therapy to standard treatment (excision and cryotherapy) when margins were positive, recurrence rates dropped down to 0% in patients who completed treatment [10]. Brachytherapy has been described for involved deep margins, but is not commonly available [15]. Medical therapy (5FU) has also been used for margin management in units that do not have access to cryotherapy, reporting recurrence rates of 11% in the treatment group vs 36% in the placebo group [12]. Excision biopsy was the main management option used in our study (*n* = 107, 94%), which means that the majority of patients had tumours that occupied four or less clock hours of the limbus. We used 4 mm margins during the excision and double cryotherapy to the limbus and free conjunctival edge. Despite this, 36% of tumours had positive margins on histology, similar to previous reports (9–43%) [11–13]. These patients had one cycle of 5FU once the epithelium had healed as an additional adjuvant. With this management approach we had a recurrence rate of 0.9% (1/107) in the surgical group. 5FU is inexpensive, freely available and well tolerated, making it a good adjuvant modality for positive deep and radial margins. Complications from surgery were minimal, confirming this as good management option for smaller OSSN lesions. Considering our low recurrence rate, we could consider smaller surgical margins for future studies to reduce surgical morbidity. Use of methylene blue and AS-OCT to delineate tumour margins has also been reported [16, 17]. With the high prevalence of positive margins, further research could look at these ancillary techniques to reduce this. Although the clinical benefit may be debatable with the successful use of adjuvant cryotherapy and medical therapy.

There has been an increased adoption of topical medical therapy for the primary management of OSSN. When used as primary therapy, resolution rates have been reported between 82 and 96% with recurrence rates of 8 and 11.5% [18–21]. We had a response in all patients with complete resolution in 71%, (5/7). 5FU was well tolerated with mild side effects that did not cause cessation of therapy.

Our study had a recurrence in two patients, one in each treatment group. Both were HIV positive and ARV naïve, which was not unique in this study. Future studies could investigate the presence of other infectious aetiologies as possible risk factors associated with recurrence.

The presence of OSSN bilaterally is uncommon [21, 22]. In our study there were five patients 5/109, 5%) that had bilateral disease. Most were female and all had HIV as a risk factor (Table 2). Overall, the median tumour surface area was almost 50% larger than the median size of the overall study. When reviewing the cases individually, the first eye tumours were larger. This is because three of the patients had bilateral disease at presentation and had the larger tumour excised first, and the other two patients had the second tumour discovered on follow-up. What was interesting was that three of the patients were virally supressed, but still developed bilateral disease. This highlights the possible multifactorial aetiology of OSSN and the importance of monitoring both eyes with follow-up. All had surgical excision and had no recurrence with follow-up.

Our study had a large number of patients with OSSN that were managed with a combination of surgery and topical 5FU. A limitation of our study was that some patients did not complete follow-up to 24 months. Of the 109 patients included in the analysis, 77 completed follow up of at least 24 months. This means that 29% (32/114) of study patients defaulted before this. We still had a median follow up of 24 months. We only had seven patients in the medical treatment group which limited our ability to provide any further insights into this group. We did not review the punctal dimensions before and after treatment with 5FU and therefore could not comment on the presence of punctal stenosis. Seven patients reported epiphora as a side effect of topical 5FU therapy, which could be attributed to drop surface toxicity or punctal involvement. Future studies could look at this side effect more closely. Our study is the largest report on the management and outcomes of OSSN in South Africa and provides a springboard for future research.

OSSN is the most common ocular surface tumour with a high burden of disease in the African context. This present study used a standardised management approach to show a low overall recurrence rate of 1.8% in our population. Future studies could further investigate 5FU as primary therapy.

## Summary

### What was known before

- HIV is commonly associated with OSSN in South Africa.
- Surgery is traditionally the gold standard of therapy for OSSN.
- 5FU is an effective medical therapy with a low side effect profile.

### What this study adds

- Surgical margins can still be involved microscopically, even when using 4 mm macroscopic margins during surgery.
- 5FU can be used as an adjuvant for managing both positive deep and radial surgical margins with low recurrence rates.
- A standardised management approach based on the size of the tumour results in a high rate of resolution and low recurrence rate.

### How this study might affect research, practice or policy

- Using 5FU for positive margins results in low recurrence rates and could reduce the burden of disease. This can be used for positive deep margins where brachytherapy is not available.
- Managing larger tumours with 5FU first could reduce the cost of a hospital admission and surgical morbidity for the patient.

## Supplementary information

Supplement 1

Supplement 2

Supplement 3

Supplement 4

## Data Availability

Data are available from the corresponding author on request.
