# Correction: Management and outcomes of ocular surface squamous neoplasia at a tertiary hospital, South Africa

**DOI:** 10.1038/s41433-026-04313-7

**Published:** 2026-03-02

**Authors:** Roland Höllhumer, Pamela Michelow, Elena Libhaber, Susan Williams

**Affiliations:** 1 Department of Neurosciences, Division of Ophthalmology, University of the Witwatersrand, Johannesburg, South Africa; 2 Cytology Unit, National Health Laboratory Service and Department of Anatomical Pathology, Faculty of Health Sciences, University of the Witwatersrand, Johannesburg, South Africa; 3 Health Sciences Research Office, Faculty of Health Sciences, University of the Witwatersrand, Johannesburg, South Africa

**Keywords:** Conjunctival diseases, Eye cancer

Correction to: *Eye* 10.1038/s41433-025-03926-8, published online 25 July 2025

In this article table 1 has been updated (percentages corrected, headings clarified.

**Table 1:** Demographics, signs, histology and risk factors for participants and tumours.

Demographics of Participants (n=109)	
	n
Median age in years [IQR]	45 [36 – 53]
Sex (%)	
Male	50 (46)
Female	59 (54)
Race (%)(n=109)	
Black African	106 (97)
Mixed Race	3 (3)
Associated Risks of Participants (n=109)	
Declined HIV testing	3 (3)
HIV positive (%)	80 (73.4)
Tobacco use (%)	21 (19)
Median hours of sun exposure [IQR]	3 (2-8)
Ocular trauma (%)	3 (3)
Chronic ocular surface inflammatory disease (%)	2 (2)
Petroleum exposure (%)	1 (1)
Median vitamin A levels^#^ [IQR]	1.64 [1.43 – 2.11]
Hepatitis B (%)	7 (6)
Hepatitis C (%)	0
Signs of Tumours (n=114)	
Mass location (%)	
Epicentre	
Conjunctiva	56 (49)
Limbus	58 (51)
Cornea	
Quadrants (%)	
Nasal	95 (83)
Temporal	22 (19)
Superior	3 (3)
Inferior	8 (7)
Clinical features (%)	
Vascularised	100 (88)
Elevated	101 (89)
Leukoplakia	61 (54)
Pigmented	58 (51)
Mild (0-33%)	24 (41)
Moderate (34-66%)	16 (28)
Severe (67-100%)	18 (31)
Morphology (%)	
Fibrovascular	14 (12)
Nodular	4 (4)
Diffuse	2 (2)
Placoid	95 (83)
Leukoplakic	60 (53)
Gelatinous	35 (31)
Papilliform	10 (9)
Median surface area, mm^2^ [IQR]	20 [9 -29]
Median limbal clock hours involved [IQR]	2 [1 -3]
Tumour thickness (%)	
<1.5mm	14 (12)
=1.5mm	82 (72)
>1.5mm	18 (16)
Scleral fixation on examination (%)	13 (11)
Histology of Tumours (n=114)	
CIN 1	35 (31)
CIN2	28 (25)
CIN 3	33 (29)
CiS	5 (4)
SCC	13 (11)

*IQR* interquartile range, *CIN* conjunctival intra-epithelial neoplasia, *CiS* squamous cell carcinoma in-situ, *SCC* squamous cell carcinoma, *HIV* human immune-deficiency virus.

Vitamin A, hepatitis B and hepatitis C were done for 66 cases.

^#^All vitamin A levels below normal had CRP reviewed to exclude a false low level due to the acute phase of an infection (none had this). Normal range for vitamin A is 1.05-2.8 μmol/L.

In the text percentages from table 1 edited where needed and “Tumour vs patient” edited in manuscript to read more clearly.

The original article has been corrected.

