# Supplementary material for: Management and outcomes of ocular surface squamous neoplasia at a tertiary hospital, South Africa
Source: Eye (Lond). 2025 Jul 25;39(14):2713–9. doi: 10.1038/s41433-025-03926-8 (PMC12446432; doi:10.1038/s41433-025-03926-8)
Supplement: Supplementary file 1 — Supplement 1 [file 41433_2025_3926_MOESM1_ESM.docx]

**Supplement 1:** Flow diagram of conjunctival mass recruitment for the study

Assessed for eligibility (n=182)

Included in the study (n=182)

Excluded (n=21)

Defaulted after biopsy or

Less than 3 months follow-up

Medical management approach (n=10)

Surgical management approach (n=125)

Clinically Benign (n=48)

Clinically OSSN (n=134)

Biopsy (n=182)

Benign on histology (n=47)

OSSN on histology (n=135)

Follow-up (n=135)

Included in this report (n=114)

Surgical management approach (n=107)

Medical management approach (n=7)
