# Supplementary material for: Management and outcomes of ocular surface squamous neoplasia at a tertiary hospital, South Africa
Source: Eye (Lond). 2025 Jul 25;39(14):2713–9. doi: 10.1038/s41433-025-03926-8 (PMC12446432; doi:10.1038/s41433-025-03926-8)
Supplement: Supplementary file 2 — Supplement 2 [file 41433_2025_3926_MOESM2_ESM.docx]

**Supplement 2:** Management approach used in this study

Adjuvant

Clinical suspicion of OSSN

≤ 4 limbal clock hours

≤ 15mm basal diameter

> 4 limbal clock hours

≤15mm basal diameter

Recurrences

Surgical excision with 4mm margins and cryotherapy

Corneal involvement

Scleral invasion

Lamellar sclerectomy

Radial and/or deep margins involved

Observe for recurrence for 24 months

1 cycle 5FU

5FU cycles

Resolution

Surgical excision

Amenable to local resection

Enucleation

Exenteration

Brachytherapy

Surgery

No

Yes

Yes

No

No

Yes

Alcohol assisted epitheliectomy
