# Supplementary material for: Management and outcomes of ocular surface squamous neoplasia at a tertiary hospital, South Africa
Source: Eye (Lond). 2025 Jul 25;39(14):2713–9. doi: 10.1038/s41433-025-03926-8 (PMC12446432; doi:10.1038/s41433-025-03926-8)
Supplement: Supplementary file 3 — Supplement 3 [file 41433_2025_3926_MOESM3_ESM.docx]

**Supplement** 3**:** Demographics, risk factors and tumour characteristics for patients with bilateral disease.

| **Demographics (n=5)** | |
| --- | --- |
| **Mean age in years (SD)** | 42 (9.7) |
| **Sex (n=5)** |  |
| **Male** | 1 |
| **Female** | 4 |
| **Race (n=5)** |  |
| **Black African** | 5 |
|  |  |
| **Associated Risks (n=5)** | |
| **HIV positive** | 5 |
| **Tobacco use** | 1 |
| **Mean hours of sun exposure (SD)** | 1.8 (1.1) |
| **Ocular trauma** | 0 |
| **Chronic ocular surface inflammatory disease** | 0 |
| **Petroleum exposure** | 0 |
| **Mean vitamin A levels^#^ (SD)** | 2.1 (1.1) |
| **Hepatitis B** | 0 |
| **Hepatitis C** | 0 |
|  |  |
| **Signs (n=10)** | |
| **Median surface area, mm^2^ [IQR]** | 38 [4 – 42.5] |
| **Median limbal clock hours involved [IQR]** | 1 [1 -3] |
| **Tumour thickness** |  |
| **<1.5mm** | 8 |
| **=1.5mm** | 1 |
| **>1.5mm** | 1 |
|  |  |
| **Outcomes (n=10)** | |
| **Recurrence** | 0 |

IQR: interquartile range

CIN: conjunctival intra-epithelial neoplasia

CiS: squamous cell carcinoma in-situ

SCC: squamous cell carcinoma

HIV: human immune-deficiency virus

Vitamin A, hepatitis B and hepatitis C were done for 3 participants

# All vitamin A levels below normal had CRP reviewed to exclude a false low level due to the acute phase of an infection (none had this). Normal range for vitamin A is 1.05-2.8umol/L.
