# Supplementary material for: Management and outcomes of ocular surface squamous neoplasia at a tertiary hospital, South Africa
Source: Eye (Lond). 2025 Jul 25;39(14):2713–9. doi: 10.1038/s41433-025-03926-8 (PMC12446432; doi:10.1038/s41433-025-03926-8)
Supplement: Supplementary file 4 — Supplement 4 [file 41433_2025_3926_MOESM4_ESM.docx]

**Supplement 4:** Complications and side effects of treatment

| **Surgery (n=107)** | | **5-Fluorouracil (n=7)** | |
| --- | --- | --- | --- |
|  | **n** |  | **n** |
| Pyogenic granuloma | 0 | Epiphora | 7 |
| Cicatrisation | 1 | Conjunctival injection | 3 |
| Limbal stem cell deficiency | 0 | Foreign body sensation | 2 |
| Persistent epithelial defect | 1 | Lid swelling | 2 |
|  |  | Superficial punctate erosions | 1 |
|  |  | Cessation of 5FU due to side effects | 0 |
